# Principles of Rational COVID-19 Therapy in Pediatrics

**DOI:** 10.3390/jcm12144731

**Published:** 2023-07-17

**Authors:** Oksana D. Nemyatykh, Marina A. Maistrenko, Dina D. Demchenko, Igor A. Narkevich, Sergey V. Okovityi, Vladimir N. Timchenko

**Affiliations:** 1 Department of Management and Economics of Pharmacy, Faculty of Pharmacy, Saint-Petersburg State Chemical Pharmaceutical University, Ministry of Health of Russia, Prof. Popova Str., 14 Lit. A, 197376 Saint-Petersburg, Russia; 2 Department of Management and Economics of Pharmacy, Faculty of Pharmacy, Ryazan State Medical University Named after Academician I.P. Pavlov, Ministry of Health of Russia, Vysokovoltnaya Str., 9, 390026 Ryazan, Russia; 3 Department of Pharmacology and Clinical Pharmacology, Faculty of Pharmacy, Saint-Petersburg State Chemical Pharmaceutical University, Ministry of Health of Russia, Prof. Popova Str., 14 Lit. A, 197376 Saint-Petersburg, Russia; 4 Department of Infectious Diseases in Children Named after Prof. M.G. Danilevich, Faculty of Pediatrics, Saint-Petersburg State Pediatric Medical University, Ministry of Health of Russia, Litovskaya Str., 2, 194100 Saint-Petersburg, Russia

**Keywords:** COVID-19, pediatrics, drug provision, treatment

## Abstract

The purpose of this review was to conduct a comparative assessment of the concepts of therapy for pediatric patients with COVID-19 in the framework of global clinical practice. A structural analysis of the range of drugs and treatment strategies in the context of etiotropic, pathogenetic, and symptomatic therapy has shown that in the global context and in real clinical practice, the etiotropic-pathogenetic approach based on information about the effectiveness of individual medical technologies prevails today. It has been established that eight international nonproprietary/grouping names are present in international practice as means of etiotropic therapy for pediatric patients with COVID-19, and 18 positions are used for pathogenetic therapy. In terms of frequency of occurrence, the leading positions are occupied by remdesivir and the combination of nirmatrelvir with ritonavir, as well as dexamethasone and tocilizumab. The paper emphasizes the relevance of research in the field of evaluating the effectiveness of individual treatment regimens as well as the analysis of the delayed consequences of pathology suffered in childhood under the conditions of using various approaches to pharmacotherapy.

## 1. Introduction

The coronavirus infection caused by SARS-CoV2 marked another milestone in healthcare and a new stage in improving medical care in pediatrics. In observations conducted in different countries in the first months of the pandemic, children usually accounted for up to 2% of laboratory-confirmed cases of SARS-CoV-2 infection, at least in the early stages of the COVID-19 pandemic [1,2,3,4,5]. 

WHO data indicate that children under the age of 18 account for approximately 8.5% of reported cases, usually with a mild form of the disease [5,6,7]. Among the age-disaggregated cases reported to WHO from 30 December 2019 to 13 September 2021, cases among children under the age of 5 years accounted for 1.8% of cases and 0.1% of deaths worldwide. Cases among children aged 5 to 14 years accounted for 6.3% of cases and 0.1% of deaths worldwide, and cases among older adolescents and young people aged 15 to 24 years accounted for 14.5% of cases and caused 0.4% of deaths worldwide [8,9].

Most studies show that the COVID-19 pandemic affects slightly more boys than girls; however, there are no significant differences in gender. With the accumulation of scientific data and the formation of an evidence base, the health systems of individual States have introduced and periodically reviewed clinical recommendations and improved treatment strategies. The versatility of pharmacotherapy approaches, as well as the diversity of the portfolio of medicines used in the treatment of pediatric patients with COVID-19 in conditions of high virulence of SARS-CoV-2, emphasize the relevance and timeliness of research in the field of medical technology expertise and the improvement of drug provision for children with the indicated infectious pathology [5,10].

The purpose of this review was to conduct a comparative assessment of the concepts of therapy for pediatric patients with COVID-19 in the framework of global clinical practice. The paper presents the results of the evaluation of the concepts of pharmacotherapy for pediatric patients with COVID-19 in international practice. The information base consisted of data extracted from open sources such as PubMed, Cochrane, and eLibrary until January 2023 by targeted searches using the reference words “COVID-19” “children”, “multisystem inflammatory syndrome” and “epidemiology of coronavirus in pediatrics”. The search was conducted in English and Russian and was limited to people. A manual reference search of the included studies was performed to avoid systematic selection errors. The inclusion criteria were any published materials on persons <18 years of age with COVID-19. After the duplicates were removed, 40 articles met the inclusion criteria. The content analysis of clinical recommendations of the North American, Western European practices and approaches of the EurAsEC was carried out on the basis of WHO recommendations “Medicines for the prevention of COVID-19: a life guide”, as well as the UK clinical recommendations “COVID-19-guidelines for the management of children admitted to hospital and for the treatment of non-hospitalized children at risk of serious illness” of the Royal College of Pediatrics and Child Health; “A brief guide to COVID-19: COVID-19 Management” of the National Institute of Health and Excellence); USA “Information on Clinical Care for COVID-19” of the US Centers for Disease Control and Prevention, “COVID-19 Treatment Guidelines” of the National Institute of Health, Russian Federation “Temporary guidelines. Prevention, diagnosis and treatment of a new coronavirus infection (COVID-19)”, versions 1–17; “Clinical protocol for the treatment of children with a new coronavirus infection (COVID-19) undergoing inpatient treatment in medical organizations of the state healthcare system of the city of Moscow”, versions 1–2; “Methodological recommendations of the Ministry of Health of the Russian Federation”, “Features of clinical manifestations and treatment of the disease caused by a new coronavirus infection (COVID-19) in children”, versions 1–2; Republic of Belarus “Recommendations (temporary) on the specifics of providing medical care to patients under the age of 18 with COVID-19 infection”, Republic of Kazakhstan “Clinical Protocol for the diagnosis and treatment of COVID-19 coronavirus infection in children of the Ministry of Healthcare of the Republic of Kazakhstan”.

## 2. Clinical Manifestations, Risk Factors and Features of the Course of COVID-19 in Pediatric Patients

The clinical picture of the course of pathology under the conditions of exposure to the virus in the child’s body has its own characteristics, both in terms of symptoms and in terms of assessing potential risks.

The first severe case of childhood infection reported in Wuhan, China, started with gastrointestinal symptoms, had no obvious early respiratory manifestations, but rapidly progressed to acute respiratory distress syndrome [11,12].

Cases of the disease in children after the appearance of the Omicron variant increased significantly (from <2% at the beginning of the pandemic to 25% from 27 January to 3 February 2022) [13]. In Sweden, in the first week of January 2022, four children with COVID-19 were hospitalized with seizures in the pediatric department in Örebro. At that time, the Omicron variant accounted for more than 98% of COVID-19 cases in the country. Three children tested positive for the virus, and one had a clinical form of COVID-19. None of the four children had a history of epilepsy or febrile seizures. J.F. Ludvigsson believes that seizures may be a sign of the Omicron variant in children [13,14,15].

The most common symptoms in children are fever, an unproductive cough, and possible signs of intoxication (myalgia, nausea, and weakness). Some patients have a sore throat, nasal congestion, and symptoms of gastrointestinal tract damage (abdominal pain, diarrhea, and vomiting) [16,17]. Thus, in the ISARIC-WHO-CCP study conducted on 651 patients under the age of 19 with laboratory-confirmed SARS-CoV-2 who were registered in the period from 17 January to 3 July 2020 in 138 hospitals in England, Scotland, and Wales, three clusters of signs were identified [18,19]:

− Respiratory cluster, in which children show separate respiratory episodes of coughing, other symptoms from the upper and lower respiratory tract, and fever;
− Systemic muco-cutaneous-intestinal clusters are manifested by headache, myalgia, gastrointestinal symptoms, lymphadenopathy, fatigue, rash, and conjunctivitis;
− A rarer cluster of neurological signs, especially seizures and confusion.

Typical signs of the disease have evolved over time as new variants of the virus have emerged. Of the symptoms described later, one can note such things as a spotted rash (as with chickenpox), neurological complications (Guillain–Barre syndrome, strokes, polyneuropathies, including rapidly transient ones), as well as psychiatric complications (delirium, followed by depression, increased anxiety, insomnia, and long-term consequences of post-traumatic stress) [20,21,22,23]. 

It was found that the clinical picture of COVID-19 in children depends on the age group. For example, children under the age of 9 most often have fever (46%), cough (37%), headache (15%), diarrhea (14%), and a sore throat (13%). While older children aged 10–19 years are more likely to have symptoms similar to COVID-19 in adults, with headache (42%), cough (41%), fever (35%), myalgia (30%), sore throat (29%), and shortness of breath (16%) [14,15].

With the clinical manifestation of COVID-19 in children, some manifestations are significantly less common or significantly more common than in adults; some symptoms are described in adults but not in children; and others, on the contrary, are described in children but not found in adults. For example, children rarely have such clinical symptoms as rhinorrhea, wheezing, and general malaise. However, diarrhea in pediatric patients with COVID-19 infection is more common than in adults [24,25,26].

To date, very little data has been recorded on the presence in children with the disease of such common symptoms in adults as anosmia/hyposmia (in adults, it is attributed to pathognomonic symptoms), conjunctivitis, and acute kidney damage. At the same time, there are specific manifestations of the disease in children, namely “COVID Fingers”. Against the background of the absence of other symptoms of the disease in children in Spain and the USA, cases of soreness of the fingers or individual phalanges with signs of cutaneous vasculitis, superficially similar to frostbite, have been described. In May 2020, new publications appeared on the presence of this sign in Italy and Spain, and systematic data in the USA allow this symptom to be recommended as pathognomonic for the diagnosis of COVID-19 in children, even in laboratory-unconfirmed cases [24,27,28].

It is generally recognized that concomitant pathology increases the risk of hospitalization or severe manifestations of the disease. British scientists conducted a meta-analysis of data on children hospitalized with COVID-19 for the period from 1 January 2020 to 21 May 2021. The risk of hospitalization in the intensive care unit (ICU) is higher for children with one chronic disease than for children without concomitant pathology. At the same time, the risk increases significantly as the number of chronic diseases increases [29].

To date, a list of conditions has been formed that are considered risk factors for severe COVID-19 in children:

− High body mass index (obesity);
− Severe pre-existing respiratory disease (asthma, COPD);
− Complex genetic or metabolic conditions associated with concomitant diseases (diabetes mellitus and impaired glucose tolerance, heart and vascular diseases);
− Multiple congenital anomalies [19,30,31,32].

Clinicians also include age (up to 1 year and 10–14 years) and non-European races as risk factors [30].

In pediatric practice, there are three stages of COVID-19 that characterize clinical manifestations at different periods of time after infection. At the first stage of the disease, which lasts for 3–7 days, the virus replicates. The SARS-CoV-2 virus can initiate a pathological response of the immune system, which in some patients reaches the level of a cytokine storm. Inadequate responses of the immune system lead to pathological activation of the hemostasis system. This is the second stage of COVID-19, which lasts about 7 days. The third stage of COVID-19 occurs approximately 14–15 days after the onset of the disease. This stage is characterized either by the patient’s recovery with a favorable course of the disease or by the development of complications with an unfavorable course [16].

It is worth noting that some children who have undergone a new coronavirus infection, COVID-19, may develop multisystem inflammatory syndrome (MIS-C). This syndrome is also called pediatric multisystem inflammatory syndrome and is temporarily associated with SARS-CoV-2. The syndrome was first described in healthy children with severe inflammation and signs of Kawasaki disease who were identified as having had a current or recent SARS-CoV-2 infection [31]. MIS-C corresponds to the postinfectious inflammatory syndrome associated with SARS-CoV-2. Most patients with MIS-C have serological signs of previous SARS-CoV-2 infection, but only some patients showed a positive result for SARS-CoV-2 upon admission [33,34,35,36].

The peak of the population incidence of MIS-C is about 4 weeks behind the peak of acute pediatric hospitalizations associated with COVID-19 [32,37,38]. MIS-C is caused by metabolic disorders in conditions of prolonged persistence of the virus in the body.

MIS-C is manifested by fever (>38.0 °C for ≥24 h), laboratory signs of inflammation (more than one violation in the following indicators: increased levels of C-reactive protein, fibrinogen, procalcitonin, D-dimer, ferritin, lactate dehydrogenase, or interleukin (IL)-6; increased erythrocyte sedimentation rate or the number of neutrophils; or decrease in the number of lymphocytes or albumin level) and clinical signs of a serious disease requiring hospitalization, with multisystem (i.e., >2) organ damage (heart, kidneys, respiratory organs, blood changes, gastrointestinal tract, skin, central nervous system) [32,39].

Patients with MIS-C are often in critical condition, and up to 80% of children need hospitalization in the ICU. The registered mortality rate in the USA for hospitalized children with MSW ranges from 1% to 2%. Currently, studies are continuing to study the long-term consequences of this syndrome [39,40].

The literature also describes such a course of SARS-CoV-2 infection in children as prolonged COVID-19. Italian scientists equate it with MIS-C; however, these conditions presumably have different etiologies. Studies concerning this manifestation are quite heterogeneous; they depend on the level of childhood morbidity in the country and on the symptoms described by parents. In official international sources, the protracted COVID-19 is not described, but there is such a thing as post-COVID. Long-term COVID includes both post-acute COVID-19 and post-ovoid syndrome. According to an international study, the duration of prolonged COVID with the preservation of some symptoms is possible up to 6–7 months [41,42].

A review of clinical practice in the field of the problem under study gives grounds to conclude that the severity of the patient’s condition is determined by the severity of clinical symptoms. To date, it has been established that a severe course is observed on average in 1% of cases. More than 95% of all cases of the disease vary from an asymptomatic course to clinical manifestations of mild and moderate severity [17,17,22,32].

WHO identifies three forms of the disease (mild, severe, and critical) [32]. The critical form of COVID-19 is determined by the criteria of acute respiratory distress syndrome (ARDS), sepsis, septic shock, or other conditions that usually require life-sustaining therapy, such as artificial ventilation (invasive or non-invasive) or vasopressor therapy. Indicators of the severe form of COVID-19 are:

- Oxygen saturation < 90% in room air;
- Signs of pneumonia;
- Signs of severe respiratory failure (very strong chest retraction, grunting, central cyanosis, or the presence of any other common dangerous sign, including inability to breastfeed or drink, lethargy, convulsions, or a decrease in consciousness) [43,44,45,46,47,48,49,50,51,52].

The non-severe form of COVID-19 is identified as the absence of any criteria for the severe or critical course of COVID-19.

In Russia, they focus on five working criteria for the severity of the COVID-19 course (asymptomatic course, mild course, moderate course, severe course, and critical course) [31,53].

The critical form of COVID-19 (extremely severe course) is determined by the occurrence of acute respiratory distress syndrome (ARDS), multisystem inflammatory syndrome (develops against the background of COVID-19 or after 3–4 weeks), hypercoagulation, DIC syndrome, and hemophagocytic syndrome (HFS).

Severe form of COVID-19 ARVI (fever, cough) at the beginning of the disease, which may be accompanied by symptoms from the gastrointestinal tract (diarrhea). The disease usually progresses within 7 days; there are signs of respiratory failure (shortness of breath with central cyanosis) and oxygen saturation of the blood <93%. On the X-ray and computed tomography (CT) of the chest organs, there are signs of lung damage typical of severe or critical viral interstitial lung damage (CT3-4).

The moderate form differs from the severe one by the absence of obvious signs of respiratory insufficiency (shortness of breath) and hypoxemia; blood oxygen saturation is >93%. A chest CT shows minor changes in the lungs, typical of mild or moderate viral lung damage (CT1-2). Also, the symptoms of a moderate form of the severity of the disease are fever >38.0 °C and cough (mainly dry, unproductive).

With a mild form of COVID-19 and an increase in body temperature of less than 38.0 °C, symptoms of intoxication (weakness, myalgia) and damage to the upper respiratory tract (cough, sore throat, nasal congestion) are usually observed. During examination, changes in the oropharynx are noted; there are no auscultative changes in the lungs. In some cases, there may be no fever, only gastrointestinal symptoms (nausea, vomiting, abdominal pain, and diarrhea), or only skin rashes observed. The oxygen saturation of the blood is greater than 95%.

In the asymptomatic form, clinical manifestations of the disease are completely absent, and only SARSCoV-2 RNA is detected in the laboratory.

The analysis of approaches to the gradation of the severity of pathology in children makes it possible to differentiate the features of international and national practices. It is worth noting that the classifications do not contradict each other. At the same time, WHO offers a single classification for children and adults. In the Russian recommendations for pediatrics, the classification of the severity of COVID-19 in children is highlighted separately (Table 1).

The analysis of the presented data allows us to conclude that the disease in childhood occurs mainly in a mild and asymptomatic form. However, children under 1 year and adolescents have a slightly increased risk of severe COVID-19. Chronic concomitant diseases also complicate the course of infectious pathology. The delayed manifestations of infectious pathology in pediatrics cause special alertness.

## 3. International Approaches to COVID-19 Pharmacotherapy in Children

It has been established that antiviral drugs, immunoglobulins, recombinant human monoclonal antibodies of the IGG1 class to ARVI-cov-2, glucocorticoids, preparations of monoclonal antibodies inhibiting IL-6 and IL-1ß receptors, anticoagulants, antiplatelet agents, as well as drugs that improve the patient’s condition, are used in the treatment of pediatric patients with COVID-19 in international practice, including antipyretic, antitussive, mucoactive, bronchodilators, and decongenants.

Unfortunately, there is currently no evidence base for antiviral drugs used in the context of the progression of COVID-19 in children, which is appropriately reflected in the multi-vector approaches to pharmacotherapy in the framework of international practice [43,44,45,46,47,48,49,50,51,52].

Remdesivir is the only drug approved by the FDA for use in pediatric practice for children over the age of 28 days and weighing more than 3 kg. At the same time, the drug is approved for use in pediatric practice in the USA and Belarus, in contrast to WHO recommendations [30,39,54,55]. It is noteworthy that scientists from China consider the drug insufficiently studied, and British pediatricians have raised the age threshold for taking remdesivir to 12–17 years [19,56]. In Russia, remdesivir has been recommended since 2021 for the treatment of children older than 12 years and weighing more than 40 kg in hospital treatment [53].

The combination of nirmatrelvir and ritonovir is approved for use in pediatric practice as an etiotropic therapy in patients with a high risk of severe disease over the age of 12 years and a body weight of at least 40 kg. The use of this drug in mild to moderate severity in people at high risk of severe disease is recommended by the Centers for Disease Control and Prevention of the United States (CDC), the Royal College of Pediatrics and Child Health (RCHCH), the National Institute of Health and Care Excellence of the United Kingdom (NICE), and the Ministry of Health of the Republic of Belarus [19,39,54,57].

Umifenovir and recombinant interferon α-2β are recommended for the prevention and treatment of COVID-19 in children by Russian infectious diseases specialists [16,31,43,44,45,46,47,48,49,50,51,52,53,58,59,60,61,62,63,64]. It is worth noting that the tactics of pharmacotherapy based on the use of interferon contradict the recommendations of the WHO and CDC, which defend a categorical position in the absence of an evidence base for the use of the designated drug [39,55].

Intravenous immunoglobulins (IVIG) are recommended for use in the development of multisystem inflammatory syndromes in Russia, China, and the USA [16,30,31,39,53,56]. The drugs are usually used in the case of a severe and critical course of the disease in pediatric patients in combination with acetylsalicylic acid and glucocorticosteroids. Attention is drawn to the fact that molnupiravir is contraindicated for use in pediatric practice due to its potential effect on bone and cartilage growth [30,31,39,40,54,58].

Currently, from the group of recombinant human monoclonal antibodies of the IGG1 class to SARS-CoV-2, sotrovimab is used in pediatric practice in Russia and the UK [19,53]. The Russian recommendations also include combinations of bamlanivimab + etesevimab and kazirivimab + imdevimab. At the same time, the positions of WHO and the USA are opposite due to the low efficiency of the indicated combinations under the conditions of the introduction of Omicron subvariants into the body [30,55].

At the end of 2022, a combination of tixagevimab and cilgavimab will be introduced to the global market. The drug is the only representative of recombinant human monoclonal antibodies against SARS-CoV-2 that can be recommended for use as a pre-contact prophylaxis. The US National Institute of Health recommends it to patients over 12 years of age and weighing at least 40 kg when the total frequency of insensitive subvariants is ≤90% [30]. The WHO concept of the drug is the opposite due to inefficiency with new strains of the virus [55]. It should be noted that these drugs are not registered on the territory of the Russian Federation, and their use is possible only if there is a permit for temporary treatment.

It is noteworthy that despite the absence of pharmacoeconomical data specific to neutralizing monoclonal antibodies (combinations of drugs tixagevimab + cilgavimab, kasirvimab + imdevimab), clinical studies have shown a reduction in the risk of hospitalization or mortality, as well as a shorter time before the disappearance of symptoms and/or clinical recovery in patients treated with monoclonal antibodies against SARS-CoV-2, compared with the control group [65,66,67,68]. Consequently, this group of drugs can contribute to reducing the costs of the healthcare system, which are associated with hospitalization and transfer to the intensive care unit, as well as the significant burden on medical organizations created by severe and critical cases of COVID-19.

Among the monoclonal antibody preparations used for COVID-19 pathogenetic therapy in children, IL-6, IL-1, TNF-alpha, and anti-IFNy receptor blockers are isolated. Therapy with IL-6 receptor inhibitors in combination with glucocorticoids is prescribed at the risk of developing severe lung damage in hospitalized children. So, tocilizumab and sarilumab are recommended for use as interchangeable drugs. At the same time, it is necessary to take into account the age threshold of use, namely: in Russia, the USA, and Belarus, therapy with these drugs is possible in patients older than two years, and in the UK—older than one year [19,30,53,54]. In Kazakhstan, the dosage regimen of tocilizumab is determined by the patient’s body weight [69].

Attention is drawn to the fact that the use of IL-1ß blockers, anti-IFNy, and monoclonal antibodies to TNF-α has not been sufficiently studied in pediatric practice. Thus, according to the US National Institutes of Health and the Ministry of Health of the Republic of Kazakhstan, the use of anakinra and infliximab is allowed in patients with MIS-C [30,39,69]. The Russian recommendations indicate the possibility of using kanakinumab at a dose of 4–8 mg/kg intravenously once, as well as anakinra in combination with normal human immunoglobulin for 48 h [53].

JAK kinase inhibitors (baricitinib and tofacitinib) are allowed for use in patients older than 2 years who need respiratory support in cases of insufficient effectiveness of glucocorticoids. Unlike JAK kinase inhibitors, Bruton tyrosine kinase inhibitors (VTK) (acalabrutinib, ibrutinib) are not recommended for use in children [19,30,54,55,58].

Glucocorticoids are used in cases of progressive respiratory failure as well as in the development of MIS-C. Dexamethasone is preferred among these drugs, but methylprednisolone can be used as an alternative [19,30,53,69]. It is noteworthy that in China and Belarus, the use of hydrocortisone is possible [54,56]. The use of prednisone in the clinical protocol for the treatment of children with COVID-19 is allowed after taking dexamethasone and methylprednisolone to reduce the dose and completely cancel this group of drugs [53].

Anticoagulant therapy is caused by the development of hypercoagulation, which is observed in patients with COVID-19. Therefore, the appointment of direct parenteral anticoagulants is indicated for all children with high-risk factors for the development of thrombotic complications. In pediatric practice, the use of anticoagulants is possible in preventive and therapeutic doses under the control of activated partial thromboplastin time (APTT). For antithrombotic purposes, unfractionated heparin (NPH), low molecular weight heparins (NMH), as well as direct and indirect anticoagulants for oral administration, are used for COVID-19 infection. The priority of choosing an anticoagulant in children belongs to NMH, namely: enoxaparin, dalteparin, and nadroparin. The dosage and frequency of prescribing drugs vary depending on the age and weight of the patient, as well as on the purpose of the appointment (preventive or curative). In Russian practice, preference is given to sodium dalteparin. In severe renal insufficiency, the administration of NPH (sodium heparin) is preferable. In the USA, preventive anticoagulant therapy is recommended for hospitalized children with COVID-19 over the age of 12 years, and with a high risk of developing MIS-C, prophylactic intake of enoxaparin sodium is indicated for children older than 2 months [19,30,53,54,69].

Antibacterial drugs in children with COVID-19 are prescribed against the background of the addition of bacterial pathogens, taking into account the results of bacteriological studies. It is worth noting that the prevailing part of the options for antibacterial therapy strategies defines amoxicillin as a first-line drug for the treatment of bacterial pneumonia. In addition, in international clinical practice, the use of drugs from the groups of penicillins, cephalosporins, and macrolides is allowed. Other antibacterial drugs are used in complex cases when the patient has a risk of developing an allergic reaction, the use of the drug is limited by the age of the patient, or severe chronic diseases are recorded in the anamnesis [16,31,53,54,58,69].

With COVID-19, heart damage occurs, as a rule, in severe, critical forms, and MIS-C in 0.5% of cases. Nevertheless, in the treatment of pediatric patients with a new coronavirus infection, the administration of drugs that can lead to an elongation of the corrected QT interval and the risk of life-threatening arrhythmias (some antiarrhythmic, antibacterial, antipsychotic, antiemetic, antifungal, vasodilating drugs, and anesthetics) requires a deep and comprehensive justification [16,53].

International approaches to effective pharmacological correction involve the use of a wide range of pharmacotherapeutic groups of drugs (Table 2).

For the treatment of pediatric patients at the outpatient stage, an important factor in the choice of drugs is the presence of a high risk of a severe course of the disease. Corticosteroids and antiviral drugs (nirmatrelvir + ritonavir) are recommended for children with risk factors in the USA and the UK [19,75,76]. In Russia, children at risk are shown to use sotrovimab, bamlanivimab in combination with etesevimab, and casirvimab in combination with imdevimab; recombinant interferon alfa-2b and umifenovir are recommended for patients with mild disease [53]. In Belarus, the use of drugs on an outpatient basis is limited to symptomatic therapy [54].

For pediatric patients undergoing inpatient treatment in the USA, remdesivir or remdesivir is used in combination with dexamethasone; in cases of ineffectiveness of this therapy, convalescent plasma, baricitinib, or tocilizumab are used in age-related dosages [75,76]. In the UK, the use of sotrovimab is recommended as part of the hospital stage [19]. In Russia, initial inpatient therapy largely repeats outpatient; however, remdesivir is used instead of umifenovir. If treatment is ineffective, it is possible to introduce immunoglobulins, antibacterial drugs (according to indications), anticoagulants, glucocorticosteroids, tocilizumab, kanakinumab, or anakinra into the treatment plan [53]. Belarusian pediatrics at the hospital stage offers the use of remdesivir and nirmatrelvir in combination with ritonovair, glucocorticoids, tocilizumab, barcitinib, and anticoagulants [54].

The literature contains recommendations for the management of hospitalized pediatric patients with MIS-C, which are based on immunomodulatory (IVIG together with methylprednisolone); intensive immunomodulating therapy (anakinra, high doses of glucocorticoids, or infliximab); and antithrombotic therapy (low doses of aspirin and anticoagulant therapy) [67]. Some studies report the use of antiviral drugs and broad-spectrum antibiotics in the first days of hospitalization for children with the syndrome [68].

Therapy for long-term COVID-19 in children is currently not clearly defined. Research is underway to describe the duration and symptoms of this complication of the transferred disease. Some researchers claim that vaccination as a preventive measure may contribute to a faster resolution of long-term COVID-19. It should be noted that the prognosis for children with a long-term diagnosis of COVID-19 is usually favorable [77,78].

## 4. Conclusions

The COVID-19 pandemic has changed the perceptions of scientists and clinicians about coronavirus infections. Against the background of the high virulence of the pathogen, SARS-CoV-2 differs from previously known representatives of the Coronaviridae family in the specifics of epidemiology, the nature of the course of the infectious process, as well as the peculiarities of manifestation in the child population. The socio-economic significance of the problem of developing effective ways to localize coronavirus and limit key links in the pathogenesis of COVID-19 in children is due to high morbidity rates as well as the likelihood of complications developing against the background of infectious pathology after acute COVID-19.

Comparative analysis demonstrates that in conditions of progression of coronavirus pathology in children, the prevailing share of international nonproprietary/grouping names used in world pediatric practice within the framework of national documents is characterized by high variability.

Despite the fact that the current level of pediatric development requires drug therapy in accordance with the principles of evidence-based medicine, the treatment of children with COVID-19 both in Russia and abroad is complicated by a lack of data on the safety, clinical, and clinical-economic effectiveness of LP in the pediatric population.

Thus, the analysis of Russian and foreign approaches to the treatment of patients with COVID-19 in pediatrics allows us to conclude that there is currently no unified strategy based on the principles of evidence-based medicine in clinical medicine, which, in turn, reveals the prospects for further research in the field of evaluating the effectiveness of individual treatment regimens as well as the analysis of delayed consequences of pathology suffered in childhood under the conditions of using various approaches to pharmacotherapy.

## Figures and Tables

**Table 1:** Approaches to the identification of the severity of COVID-19 in pediatrics [31,32,53].

No	WHO Approaches	Approaches in Russian Practice
1.	Unified classification of forms and severity of the disease for all ages.	Different classifications for children and adults.
2.	3–4 forms of gravity.	5 forms of gravity.
3.	The classification does not provide. specific criteria by which the patient should be assigned to a particular form of severity.	The classification details the symptoms and results of examinations that allow the patient to be attributed to one form or another of severity.
4.	Oxygen saturation of the blood in severe form should be less than 90%.	Oxygen saturation of the blood in severe form should be less than 93%.
5.	The critical form includes “sepsis, septic shock”.	“Sepsis” and “septic shock” are prescribed as complications.

**Table 2:** Structure of the range of medicines for the treatment of COVID-19 in children in international practice [30,31,32,39,53,54,55,56,57,58,59,60,61,62,63,64,69,70,71,72,73,74].

ATC-Code	INN/Grouping Name	WHO	CDC	NIH	RCHCH/ NICE	Ministry of Health of Russia	Ministry of Health of the Republic of Belarus	Ministry of Health of the Republic of Kazakhstan
Antiviral drugs								
J05AX13	Umifenovir	NA	NA	NA	NA	R	NR	NA
J05AE30	Nirmatrelvir + ritonavir	NA	R	R	R	NA	R	NA
J05AB16	Remdesivir	NA	R	R	R	R	R	NA
J05AB18	Molnupiravir	NA	C	C	NA	NA	NA	NA
Interferons								
L03AB	Recombinant interferon-alpha	NR	NA	NR	NA	R	NA	NA
Immunoglobulins								
J06BA02	Human normal immunoglobulin	NA	NA	R	NA	R	NA	NA
Monoclonal antibodies								
Specific monoclonal antibodies against COVID-19								
J06BD05	Sotrovimab	NR	NA	NA	R	R	NA	NA
	Bamlanivimab+ etesevimab	NA	NA	NA	NA	R	NA	NA
J06BD07	Kazirivimab+ imdevimab	NR	NA	NA	NA	R	NA	NA
Non-specific monoclonal antibodies								
L04AC07 L04AC14	Tocilizumab Sarilumab	NA	NA	R	R	R	R	R
L04AC08	Kanakinumab	NA	NA	NA	NA	R	NA	NA
L04AC03	Anakinra	NA	NA	R	NA	R	NA	R
L04AB02	Infliximab	NA	NA	R	NA	NA	NA	R
L04AA37 L04AA29	Baricitinib Tofacitinib	NA	NA	R	R	R	R	NA
L01EL02 L01EL01	Akalabrutinib Ibrutinib	NA	NA	C	NA	NA	NA	NA
Glucocorticoids								
H02AB02	Dexamethasone	NA	NA	C	R	R	R	R
H02AB04	Methylprednisolone	NA	NA	R	NA	R	R	NA
D07AA03	Prednisolone	NA	NA	NA	NA	R	R	R
D07AA02	Hydrocortisone	NA	NA	NA	NA	NA	R	NA
Anticoagulants								
B01AB04	Dalteparin	NA	NA	R	R	R	R	NA
C05BA53	Heparin	NA	NA	NA	NA	R	NA	R
B01AB05	Enoxaparin	NA	NA	R	NA	NA	R	NA
B01AB06	Nadroparin	NA	NA	NA	NA	NA	R	NA
Antiplatelet agents								
N02BA01	Acetylsalicylic acid	NA	NA	R	NA	NA	NA	NA

**Note:** INN—international nonproprietary name, WHO—World Health Organization, NIH—National Institutes of Health, RCHCH—Royal College of Paediatrics and Child Health, NICE—National Institute for Health and Care Excellence, Ministry of Health of Russia—Ministry of Health of the Russian Federation, Ministry of Health of the Republic of Belarus—Ministry of Health of the Republic of Belarus, Ministry of Health of the Republic of Belarus Kazakhstan—Ministry of Health of the Republic of Kazakhstan, NA—no data available, C—contraindications, R—recommended, NR—not recommended.

## Data Availability

Not applicable.
